# Regulatory Effect of Sishen Pill on Tfh Cells in Mice With Experimental Colitis

**DOI:** 10.3389/fphys.2020.00589

**Published:** 2020-06-05

**Authors:** Xue-Ke Liu, Hai-Mei Zhao, Hai-Yan Wang, Wei Ge, You-Bao Zhong, Jian Long, Duan-Yong Liu

**Affiliations:** ^1^Department of Postgraduate, Jiangxi University of Traditional Chinese Medicine, Nanchang, China; ^2^College of Traditional Chinese Medicine, Jiangxi University of Traditional Chinese Medicine, Nanchang, China; ^3^Party and School Office, Jiangxi University of Traditional Chinese Medicine, Nanchang, China; ^4^Department of Proctology, Affiliated Hospital of Jiangxi University of Traditional Chinese Medicine, Nanchang, China; ^5^Science and Technology College, Jiangxi University of Traditional Chinese Medicine, Nanchang, China; ^6^Pharmacology Office, Key Laboratory of Pharmacology of Traditional Chinese Medicine in Jiangxi, Nanchang, China

**Keywords:** Sishen Pill, Tfh cell, colitis, BCL-6/Blimp-1, mechanism

## Abstract

The T follicular helper T (Tfh) cells play a significant role in the pathogenesis of inflammatory bowel disease (IBD), which is regulated by the Bcl-6/Blimp-1 pathway. Some studies have suggested that regulating activation of the Bcl-6/Blimp-1 pathway should be an effective method to treat IBD. Sishen Pill (SSP) has been used frequently to treat chronic colitis. Its mechanism is related to the downstream proteins in the Bcl-6/Blimp-1 pathway. However, it is unknown whether SSP regulates the function and differentiation of Tfh cells to treat IBD. In the present study, chronic colitis was induced by dextran sodium sulfate and treated with SSP for 7 days. SSP effectively treated chronic colitis, regulated the balance between Tfh10, Tfh17 and T follicular regulatory cells, while SSP increased the Blimp-1 level, inhibited expressions of Bcl-6, T-cell costimulator, programmed death (PD)-1 and PD-ligand 1 on the surface of Tfh cells. SSP inhibited activation of BcL-6, phosphorylated signal transducer and activator of transcription (p-STAT)3, signal lymphocyte activation molecule (SLAM)-associated protein but improved Blimp-1 and STAT3 expression in colonic tissues. The results indicated that SSP regulated the differentiation and function of Tfh cells to treat IBD, which was potentially related with inhibiting the Bcl-6/Blimp-1 pathway.

## Introduction

Inflammatory bowel diseases (IBDs), including ulcerative colitis (UC) and Crohn’s disease (CD), are characterized by idiopathic chronic intestinal inflammation (Sairenji et al., 2017). Their pathogenic factors are closely related to the environment, microorganisms, genes, and immunity (Zhang and Li, 2014; Kelsen and Sullivan, 2017). In recent years, a large number of studies have found that abnormal function and differentiation of T follicular helper (Tfh) cells can lead to autoimmune diseases including IBD (Mesquita et al., 2016; Ueno, 2016).

Ten years ago, many researchers found a new subpopulation of CD4^+^ T cells, Tfh cells, localized to B cell follicles and germinal centers (GCs) (Breitfeld et al., 2000; Schaerli et al., 2000). Tfh cell differentiation is mainly divided into three phases (Crotty, 2014). The first phase is early Tfh cell differentiation, which is mainly performed by dendritic cells (DCs). Many studies have shown that high levels of T-cell receptor can lead to continuous interaction between Tfh cells and DCs (Tubo et al., 2013). The second phase is the finish of Tfh cells differentiation, which is located at the T cell and B cell accumulation area by chemokine CXC receptor (CXCR)5 expression (Webb and Linterman, 2017). The third phase is mainly located at the GC, which can highly express CXCR5 and programmed death (PD)-1, while other markers mainly include CXCR5, GL7, and inducible T-cell costimulator (ICOS). Tfh cells are located at the follicular area of the mutant B cells, secrete interleukin (IL)-21, induce expression of transcription factor Bcl-6, and promote high-affinity B cell clones (Weinstein et al., 2016). Functionally, Tfh cells are divided into Tfh1, Tfh2, Tfh10, Tfh17, Tfh21, and Tfr cell lineages, which respectively produce or express interferon (IFN)-γ, IL-4, IL-10, IL-17, IL-21, and forkhead box (FOX)P3, and these are closely related to colitis (Fazilleau et al., 2009; Crotty, 2014; Jandl et al., 2017).

It has been shown that high expression of Tfh-cell-related genes leads to development of IBD (Zhang et al., 2019). Clinical trials have shown that the balance of Tfr and Tfh cells is closely related to IBD (Wang et al., 2019b). In IBD patients, FOXP3 and IL-10 levels decrease while IL-21 level increases, which are positively correlated with the decreased ratio of Tfr/Tfh cells (Wang et al., 2017). Liu et al. demonstrated that Tfh cell population was increased in mice with dextran sulfate sodium (DSS)-induced colitis, and then reduced after the colonic damage was alleviated by probiotics (Liu et al., 2019). The results suggest that Tfh cells may become a new target for the treatment of IBD.

In China, Sishen Pill (SSP) is the classic prescription of traditional Chinese medicine (TCM) for 1,000 years, and frequently-used to treat IBD, allergic colitis, chronic colitis, irritable bowel syndrome (IBS), and so on (Wang et al., 2019a). In clinic, SSP is widely accepted by IBD patients for definite therapeutic effect, little adverse reaction, and lower recurrence rate (Lu et al., 2011). In previous studies, SSP was found to be effectively treat colitis in rats by inhibiting signal transducer and activator of transcription (STAT)3, IFN-γ, IL-1β, IL-2, IL-6, and IL-17 expressions, and increase IL-4 and transform growth factor (TGF)-β expression (Liu et al., 2015; Zhao et al., 2019). These cytokines are associated with Tfh cells, but it is not known whether SSP can regulate Tfh cells to treat IBD. In the present study, the mechanism underlying the anti-colitis effect of SSP was investigated in DSS-induced colitis in mice.

## Materials and Methods

### Drugs

SSP (batch number 17080051) was purchased from Tongrentang Natural Medicine Co. Ltd., (Beijing, China), which was composed by *Euodia rutaecarpa* (Juss.) Benth., *Schisandra chinensis* (Turcz.) Baill, *Psoralea corylifolia* L., *Myristica fragrans* Houtt., *Ziziphus jujuba* Mill., and *Zingiber officinale* Rosc. Which were prepared into pills according to the dose ratio (100, 200, 400, 200, 200, and 200 g, ratio: 1:2:4:2:2:2, respectively). SSP contained deoxyschizandrin (72.6 μg/g), γ-schizandrin (131.5 μg/g), schizandrin (258.0 μg/g), schizandrol B (71.2 μg/g), schisantherin A (25.1 μg/g), psoralen (131.08 μg/g), isopsoralen (1293.7 μg/g), evodiamine (22.2 μg/g), and rutaecarpine (24.0 μg/g). SSP was analyzed by high-performance liquid chromatography coupled with electrospray tandem mass spectrometry (Zhang et al., 2018). DSS (molecular weight: 36,000–50,000 kDa; No. 160110) was obtained from MP Biomedicals (Santa Ana, CA, USA). Mesalazine was obtained from Jiamusi Luling Pharmaceutical Co., Ltd., Company (Jiamusi, China).

### Animals

Male BALB/C mice weighing 18–22 g (Animal Certificate No. SCXK 2006-0008) were purchased from Hunan Slake Jing da Experimental Animal Co., Ltd. (Changsha, China). All animals were housed in specific-pathogen-free conditions, with standard laboratory diet, 12-h light/dark cycle and constant room temperature, and had free access to standard diet and tap water according to the guidelines of the Animal Center. This Protocol (License No.: JZ2018-105) was approved by the Institutional Animal Care and Use Committee (IACUC) of Jiangxi University of Traditional Chinese Medicine. The mice were acclimated for 3 days according to the IACUC Animal Welfare Guidelines before formal experiments were performed. Thirty-two mice were divided into two groups: eight mice were in the normal group and the remaining mice were treated by DSS to induce colitis. The twenty-four mice were observed to have bloody stools on the fourth or fifth day after DSS treatment, which hinted the colitis was successfully induced. Twenty-four colitis mice were randomly divided into three groups: DSS: colitis without treatment; DSS + SSP: colitis treated with SSP; and DSS + 5-ASA: colitis treated with 5-aminosalicylic acid (ASA). Eight colitis mice were in every group.

### DSS-Induced Colitis

According to the previous study (Yoshihara et al., 2006), colitis was induced in BALB/C mice with 3% (w/v) DSS (molecular weight: 36,000–50,000 kDa) dissolved in deionized water drunk ad libitum (days 1–7). Fresh 3% DSS solutions were made every morning in deionized water. Control mice were given tap water.

### Therapeutic Protocols

On day 8, the DSS + SSP group was administered 2.5 g kg^−1^ SSP dissolved in physiological saline by oral gavage for 7 days; the DSS + 5-ASA group was administered 300 mg kg^−1^ mesalazine by oral gavage for 7 days; and mice in DSS and normal groups were treated with the same volume of physiological saline by oral gavage for 7 days. On day 15, all animals were sacrificed under sodium pentobarbital (50 mg/kg ip) anesthesia.

### Macroscopic Evaluation

The mice were weighed before anesthesia, and the colons were quickly removed. The colon length and weight were measured, and the colon weight index (CWI), CWI = colon weight/body weight × 100 was calculated.

### Hematoxylin and Eosin (H&E) Staining and Microscopic Evaluation

The colon was preserved in a 4% polyformaldehyde solution for 7 days, then dehydrated and embedded in paraffin, and the paraffin sections were serially sectioned at 4 μm. The tissue sections were dewaxed and rehydrated using an alcohol gradient and stained with H&E. The pathological features of the colon were observed and evaluated under a microscope. The histological grading of colitis was as described by Dieleman et al. (1998). Inflammation: none, 0 points; slight, 1 point; moderate, 2 points; and severe, 3 points. Extent: none, 0 points; mucosa, 1 point; and mucosa and submucosa, 3 points. Regeneration: no tissue repair, 4 points; surface epithelium not intact, 3 points; regeneration with crypt depletion, 2 points; almost complete regeneration, 1 point; and complete regeneration or normal tissue, 1 point. Crypt damage: none, 0 points; basal 1/3 damaged, 1 point; basal 2/3 damaged, 2 points; only surface epithelium intact, 3 points; and entire crypt and epithelium lost, 4 points. Percent involvement: 1–25%, 1 point; 26–50%, 2 points; 51–75%, 3 points; and 76–100%, 4 points.

### Enzyme-Linked Immunosorbent Assay

The colon tissue of the mice was collected, and the RIPA tissue lysate was added at a ratio of 1:10, incubated at 4°C for 1 h, homogenized by ultrasonic homogenizer for 20 min, centrifuged at 4°C at 19,375 *g* for 30 min, and the supernatant was obtained and analyzed. The levels of TNF-α (No. 88-7324-22), IL-6 (No. 88-7064-22), IL-10 (No. 88-7105-22), IL-17 (No. 88-8711-88), and IL-23 (No. 88-7230-88) (eBioscience, San Diego, CA, USA) and TGF-β1 (No. 555052) (BD Biosciences, Franklin Lakes, NJ, USA) were measured by commercial ELISA kits, and absorbance at 450 nm was read using a microplate reader (Bio-Rad, Hemel Hempstead, UK).

### Flow Cytometry

For isolation of peripheral lymphocytes, 500 μl peripheral blood were collected from each mouse and lysed by treatment with 1 ml lysing Solution (BD Biosciences, Franklin Lakes, NJ, USA) to clear red blood cells. The obtained lymphocytes were incubated with fluorescence-conjugated monoclonal antibodies in staining buffer. Eight-color flow cytometry analysis (*n* = 8) was performed on a FACSCalibur device (Becton Dickinson, Mountain View, CA, USA). The frequency of positive cells was analyzed using the program Cell Quest in two regions. The lymphocyte region was determined using granularity (SSC) and size (FSC) plots. Tfh cells were identified as a CD4^+^lineage^+^ (CXCR5^+^, ICOS^+^, FOXP3^+^, IL-10^+^, IL-17^+^, and BCL-6^+^) population, and within this group, the CD4^+^ population was assessed. The following monoclonal antibodies were used: APC-H7 anti-mouse CD4 (No. 560181) (1:200), FITC Anti-Mouse CXCR5 (No. 560577) (1:100), PE Anti-Mouse ICOS (No. 565669) (1:100), PerCP-Cy5.5 Rat Anti-Mouse FOXP3 (No. 563902) (1:100), APC Anti-Mouse IL-10 (No. 554468) (1:200), APC-Cy7 Anti-Mouse IL-17 (No. 560821) (1:200), APC Anti-Mouse PD-1 (No. 562671) (1:200), PE Anti-Mouse PD-L1 (No. 558091) (1:200), and PerCP-Cy5.5 Anti-Mouse BCL-6 (No. 563582) (1:200) (BD Biosciences, Franklin Lakes, NJ, USA). Limits for the quadrant markers were always set based on negative populations and isotype controls.

### Western Blotting

Colon specimens were homogenized and the supernatant was extracted as described above. Protein concentration was determined by classical BCA (Beyotime, Nanjing China) protein assay. Equal amounts of total protein (60–80 μg) were subjected to SDS-PAGE and transferred to a polyvinylidene fluoride membrane using a Bio-Rad Western blotting apparatus. After blocking with 5% non-fat milk or bovine serum albumin, primary antibodies were added overnight at 4°C. The antibodies were anti-GAPDH (No. 160110) (1:3,000), BCL-6 (No. ab19011) (1:1,000), p-STAT3 (No. ab131103) (1:2,000), SAP (No. ab185810) (1:2,000), Blimp-1 (No. ab7888) (1:1,000), and STAT3 (No. ab119352) (1:2,000) (Abcam, Cambridge, MA, USA). The sections were incubated with a second antibody [Goat Anti-Rabbit lgG H&L (HRP)] (No. 150077) (1:2,000–1:4,000) (Abcam) for 1 h at 37°C. The labeled protein bands were scanned with an HP Scanjet 5500 (Hewlett Packard France, Les Ullis, France).

### Statistical Analysis

All statistical analyses were performed using GraphPad Prism software, using one-way analysis of variance followed by a Tukey’s test of multiple comparisons. *p* < 0.05 indicated that the difference was significant.

## Results

### SSP Ameliorated DSS-Induced Colitis

**Figure 1:**
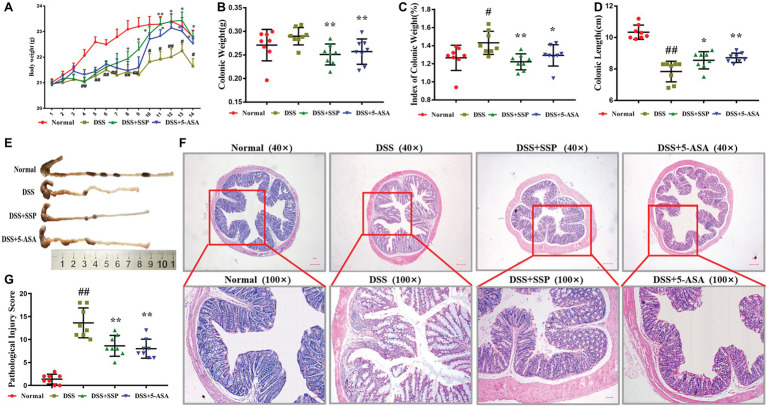
Therapeutic evaluation of Sishen Pill (SSP)-treated colitis mice. **(A)** Body weight change in every day. **(B)** Colonic weight. **(C)** Colonic weight index. **(D)** Colonic length. **(E)** Typical image of a complete colon. **(F)** Typical histological images of Hematoxylin and Eosin (H&E) staining. **(G)** Pathological damage score, Normal: health mice without treatment by dextran sulfate sodium (DSS); DSS: colitis induced by DSS without treatment; DSS + SSP: colitis treated with SSP; and DSS + 5-ASA: colitis treated with 5-aminosalicylic acid (ASA). Data were presented as means ± SEM (*n* = 8). These images are shown at the same magnification (×40 or ×100). ^#^*p* < 0.05 and ^##^*p* < 0.01 versus the normal group; ^*^p < 0.05 and ^**^p < 0.01 versus the DSS group.

DSS-induced colitis is a classic model to develop new drugs for treatment of IBD. After 4–5 days’ administration of 3% DSS, bloody stools were observed *via* the naked eye, body weight decreased, and hair was sparse and lusterless. Mice in the DSS group had significantly reduced body weight compared to the normal group (Figure 1A), while their colon length was shorter (Figures 1D,E), and the colonic weight and colon weight index were significantly increased (Figures 1B,C). Pathological observation showed ulcer formation, serious hyperemia and edema, and many inflammatory cells in the colonic mucosa (Figure 1F). The pathological damage score was increased compared with that in the normal group (Figure 1G). After 7 days’ treatment with SSP or 5-ASA, compared with the DSS group, the body weight was increased (Figure 1A) in the third day to eighth day, colonic length was restored (Figures 1D,E), and colonic weight and colonic weight index were reduced (Figures 1B,C). Pathological sections of colonic tissues from colitis mice treated with SSP and 5-ASA showed fewer ulcers, decreased inflammatory cell infiltration, and colonic epithelial cell hyperplasia (Figure 1F). The pathological damage scores of mice treated with SSP and 5-ASA were lower than in the DSS group (Figure 1G). The results showed that SSP ameliorated pathological colonic damage in DSS-induced colitis.

### SSP Inhibited IL-6, IL-23, and TGF-β1 Expression in Mice With Colitis

**Figure 2:**
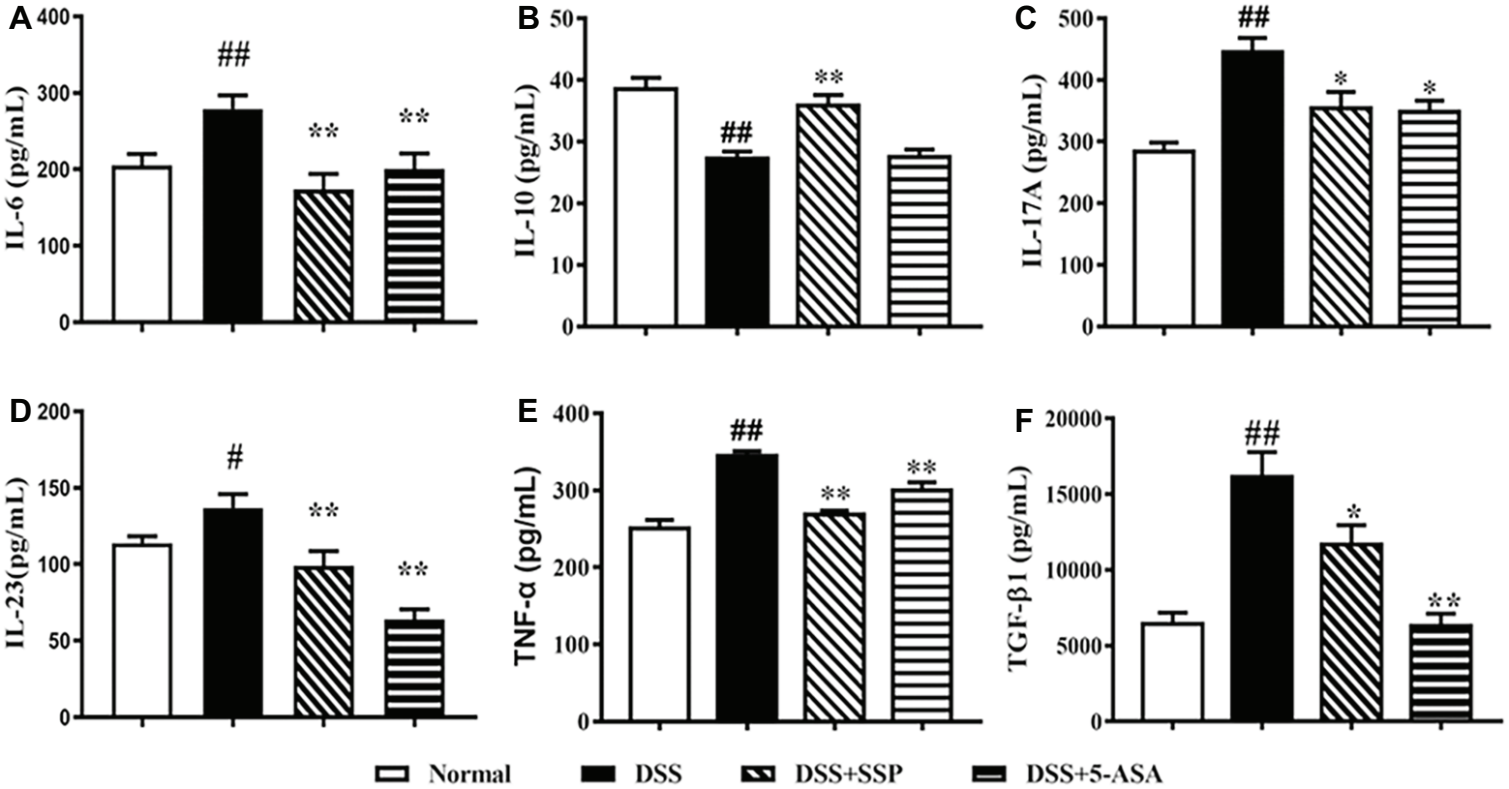
Levels of IL-6, IL-10, IL-17, IL-23, TNF-α, and TGF-β1. **(A)** IL-6. **(B)** IL-10. **(C)** IL-17. **(D)** IL-23. **(E)** TNF-α. **(F)** TGF-β1. Data were presented as means ± SEM (*n* = 8). ^#^*p* < 0.05 and ^##^*p* < 0.01 versus the normal group; ^*^p < 0.05 and ^**^p < 0.01 versus the DSS group.

The levels of IL-6 (Figure 2A), IL-17A (Figure 2C), IL-23 (Figure 2D), TNF-α (Figure 2E), and TGF-β1 (Figure 2F) in the colonic mucosa of colitis mice without treatment were higher than in the DSS + SSP and DSS + 5-ASA groups. While the IL-10 expression were increased when colitis mice were treated by SSP. The results indicated that SSP inhibited pro-inflammatory factors (as IL-6, IL-17A, IL-23, and TNF-α) and improved IL-10 (Figure 2B) expression, or restrained TGF-β1 in the colonic tissue of colitis mice.

### SSP Regulated the Quantities of Tfh10, Tfh17, and Tfr Cell in Colitis Mice

**Figure 3:**
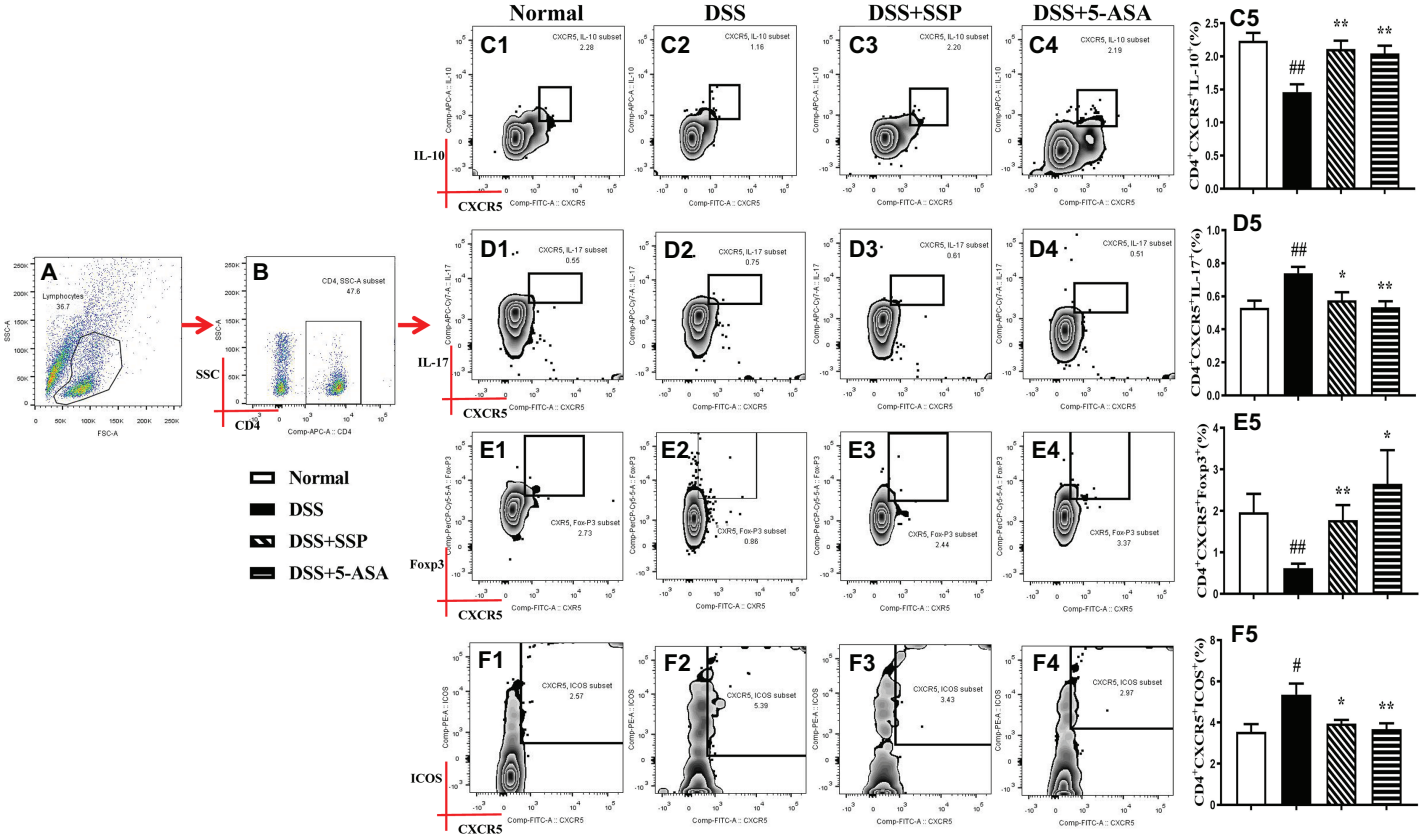
Flow cytometry to detect CD4^+^CXCR5^+^IL-10^+^, CD4^+^CXCR5^+^IL-17^+^, CD4^+^CXCR5^+^FOXP3^+^, and CD4^+^CXCR5^+^ICOS^+^ cell levels. **(A)** Total lymphocytes in peripheral blood. **(B)** Gating strategy for CD4^+^ cells. **(C)** C1–C5: CD4^+^CXCR5^+^IL-10^+^ cell levels. **(D)** D1–D5: CD4^+^CXCR5^+^IL-17^+^ cell levels. **(E)** E1–E5: CD4^+^CXCR5^+^FOXP3^+^ cell levels. **(F)** F1–F5: CD4^+^CXCR5^+^ICOS^+^ cell level. Data were presented as means ± SEM (*n* = 8). ^#^*p* < 0.05 and ^##^*p* < 0.01 versus the normal group; ^*^p < 0.05 and ^**^p < 0.01 versus the DSS group.

Tfh cells can be divided into many subgroups, including Tfh1, Tfh2, Tfh4, Tfh9, Tfh10, Tfr, and so on. Compared with the normal group, flow cytometry found that the numbers of CD4^+^CXCR5^+^IL-10^+^ (Tfh10) (Figures 3A–C) and CD4^+^CXCR5^+^Foxp3^+^ (Tfr) (Figures 3A,B,E) in the peripheral blood of mice in the DSS group were significantly decreased, while the number of CD4^+^CXCR5^+^ IL-17^+^ (Tfh17) (Figures 3A,B,D) was significantly elevated. After 7-days’ administration of SSP and 5-ASA, the numbers of CD4^+^CXCR5^+^IL-10^+^ (Tfh10) (Figures 3A–C) and CD4^+^CXCR5^+^Foxp3^+^ (Tfr) (Figures 3A,B, E5) were dramatically increased when compared with those in colitis mice without treatment. However, the number of CD4^+^CXCR5^+^ IL-17^+^ (Tfh17) (Figures 3A,B,D) was markedly decreased. The results suggested that SSP regulated the balance of the subsets of Tfh cells in colitis mice.

### SSP Optimized the Differentiation of Tfh Cells in Colitis Mice

**Figure 4:**
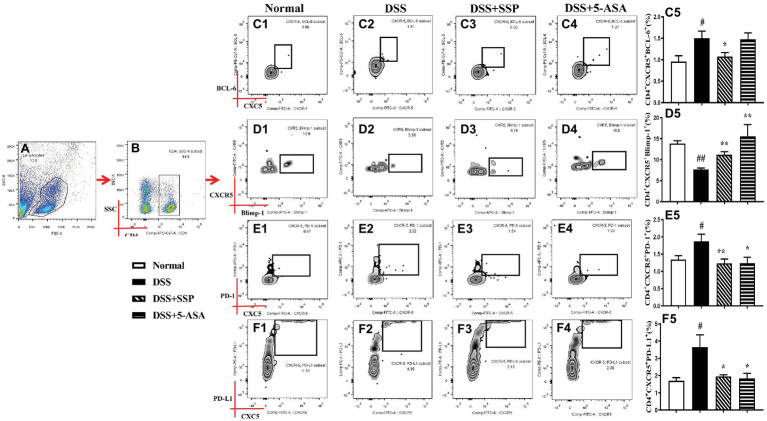
Flow cytometry to detect CD4^+^CXCR5^+^BCL-6^+^, CD4^+^CXCR5^+^Blimp-1^+^, CD4^+^CXCR5^+^PD-1^+^, and CD4^+^CXCR5^+^PD-L1^+^ cell levels. **(A)** Total lymphocytes in peripheral blood. **(B)** Gating strategy for CD4^+^ cells. **(C)** C1–C5: CD4^+^CXCR5^+^BCL-6^+^ cell levels. **(D)** D1–D5: CD4^+^CXCR5^+^Blimp-1^+^ cell levels. **(E)** E1–E5: CD4^+^CXCR5^+^PD-1^+^ cell levels. **(F)** F1–F5: CD4^+^CXCR5^+^PD-L1^+^ cell level. Data were presented as means ± SEM (*n* = 8). ^#^*p* < 0.05 and ^##^*p* < 0.01 versus the normal group; ^*^p < 0.05 and ^**^p < 0.01 versus the DSS group.

The abnormal hyperactivity of Tfh cells as a CD4^+^ T subpopulation leads to the development of autoimmune diseases including IBD, autoimmune liver disease, and rheumatoid arthritis (RA), which were induced by abnormal expressions of related signaling proteins. Compared with the normal group, the numbers of CD4^+^CXCR5^+^BCL-6^+^ (Figures 4A–C), CD4^+^CXCR5^+^ICOS^+^ (Figures 3A,B,F), CD4^+^CXCR5^+^PD-1^+^ (Figures 4A,B,E), and CD4^+^CXCR5^+^PD-L1^+^ (Figures 4A,B,F) T cells were significantly elevated, while CD4^+^CXCR5^+^Blimp-1^+^ T cells (Figures 4A,B,D) were inhibited in the peripheral blood of colitis mice in the DSS group. In contrast with mice in the DSS group, the numbers of CD4^+^CXCR5^+^ BCL-6^+^ (Figures 4A–C), CD4^+^CXCR5^+^ICOS^+^ (Figures 3A,B,F), and CD4^+^CXCR5^+^PD-1^+^ (Figures 4A,B,E) T cells were decreased, and CD4^+^CXCR5^+^Blimp-1^+^ T cells (Figures 4A,B,D) were significantly increased after colitis was treated with SSP and 5-ASA for 7 days. These results indicated that SSP optimized differentiation of Tfh cells in colitis mice.

### SSP Controlled Activation of BCL-6/Blimp-1 Signaling Pathway in Colitis Mice

**Figure 5:**
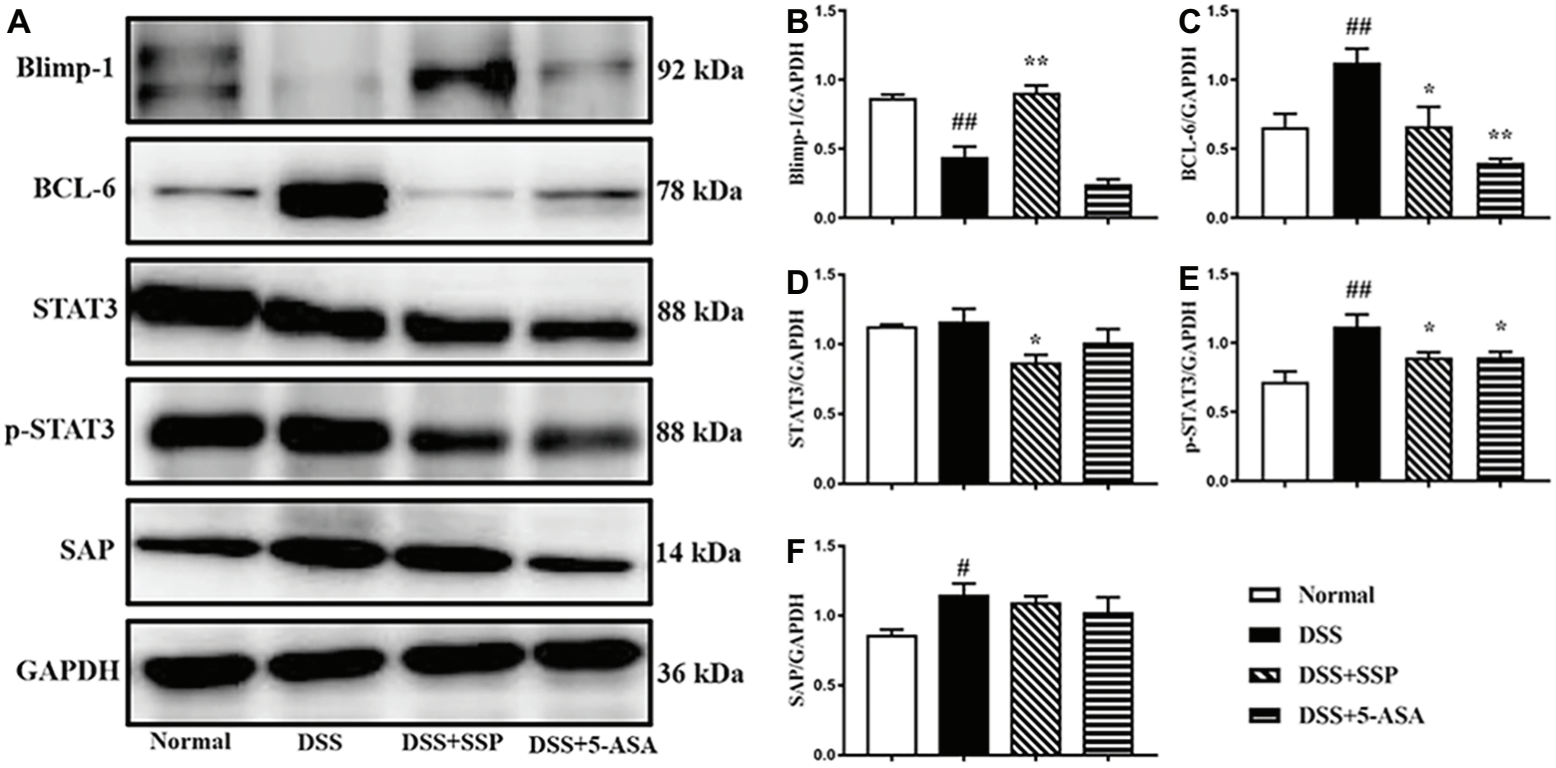
Western blot analysis of the BCL-6/Blimp-1 signaling pathway. **(A)** Western blot of Blimp-1, BCL-6, STAT3, p-STAT3, and SAP. **(B)** Quantitative analysis of Blimp-1. **(C)** Quantitative analysis of BCL-6. **(D)** Quantitative analysis of STAT3. **(E)** Quantitative analysis of p-STAT3. **(F)** Quantitative analysis of SAP. ^#^*p* < 0.05 and ^##^*p* < 0.01 versus the normal group; ^*^p < 0.05 and ^**^p < 0.01 versus the DSS group.

Through Western bolting assay, we verified the key proteins in the Bcl-6/Blimp-1 signaling pathway and found that SSP significantly inhibited expression of Bcl-6 (Figures 5A,C), STAT3 (Figures 5A,D), and p-STAT3 (Figures 5A,E) proteins and improved expression of Blimp-1 (Figures 5A,B) in colonic tissues of colitis mice. It was not obvious that SSP regulated signal lymphocyte activation molecule (SLAM)-associated protein (SAP) expression (Figures 5A,F). The results indicated that SSP inhibited BCL-6/Blimp-1 signaling pathway in colitis mice.

## Discussion

IBD is a nonspecific colonic inflammation, whose common clinical manifestations include abdominal pain, diarrhea, fecal occult blood, and hematochezia. Colonoscopy clearly shows colonic mucosal hyperemia, edema, petechia, and ulcer formation. In the present study, the colitis model was induced in BALB/C mice by DSS. The body weight of the mice began to decrease on the second day after drinking 3% DSS, and bloody stools were observed on the day 5. After SSP treatment, body weight, colon weight, and colon length of the mice were restored. Pathological observation showed crypt epithelial cell dropout, crypt destruction, and variable lymphocytic infiltration of epithelium and lamina propria in untreated colitis mice. However, after 7-days’ treatment with SSP, there was evidence of improvement in pathological colonic damage, with fewer inflammatory cells, gland irregular hyperplasia, fewer and smaller ulcers in the mucosa, and decreased colonic weight index and pathological damage score, which indicated that SSP effectively treated DSS-induced chronic colitis. At the same time, IL-6, IL-17A, IL-23, TNF-α, and TGF-β1 expressions were significantly decreased in colitis mice treated by SSP. As pro-inflammatory factors, IL-6, IL-17A, IL-23, and TNF-α are classical hallmarks of colitis (Xiao et al., 2016; Allocca et al., 2018), which induce the occurrence of colitis. And IL-17A is regard as a main product of Tfh17 cells, so high-expressed IL-17A in colitis mice hinted that elevated level of Tfh17 cells is possible phenomenon in the pathogenesis of colitis. As a classic anti-inflammatory factor, IL-10 secretion was decreased for the downregulated function of Tfh10 cells in DSS-induced colitis. TGF-β1 has both anti-inflammatory and pro-inflammatory effects in different diseases. In the present study, elevated levels of TGF-β1 in DSS-induced colitis were positively correlated with disease progression. Vitamin D can treat colitis by inhibiting TGF-β1 (Stadnicki et al., 2009; Tao et al., 2015). The elevated level of TGF-β1 in the DSS group may play a pro-inflammatory role, and was decreased after treatment, which proves that SSP inhibited expression of pro-inflammatory factors to alleviate colonic mucosal damage in experimental colitis. Mesalamine, or 5-aminosalicylic acid (5-ASA), is a mainstay of therapy for individuals with inflammatory bowel disease. In the present study, 5-ASA was selected to evaluate the therapeutic effect of SSP as control drug. As a classic chemical drug in anti-inflammatory therapy of IBD, 5-ASA can inhibit NF-κB signaling pathway to decrease inflammatory secretion, or restrain prostaglandin or leucotriene to eliminate inflammatory injury. In anti-inflammatory action, it is obvious that 5-ASA is better than SSP. However, it is fortunate that SSP has an analogous anti-inflammatory action as downregulating pro-inflammatory factors (as IL-6, IL-17A, IL-23, and TNF-α and so on) or upregulating anti-inflammatory factor (IL-10). Certainly, the effect of SSP are realized by regulating immune or apoptosis and so on.

In recent years, it has been found that Tfh cells are closely related to colitis. Due to their heterogeneity, Tfh1, Tfh2, Tfh10, Tfh17, Tfh21, and Tfr cells have different immunological functions and can be differentiated (Fazilleau et al., 2009). Therefore, in this experiment, CD4^+^CXCR5^+^IL-10^+^ (Tfh10), CD4^+^CXCR5^+^IL-17^+^ (Tfh17), and CD4^+^CXCR5^+^Foxp3^+^ (Tfr) cells were measured. The numbers of Tfh10 and Tfr cells were decreased and the number of Tfh17 cells was increased in the DSS group, which was reversed after treatment with SSP. CD4^+^Foxp3^+^Treg cells play a major role in immune homeostasis (Jung et al., 2017), and their expression is decreased (Ohno et al., 2017) in colitis mice. Tfr cells are a specialized subset of Treg cells with high expression of CXCR5^+^ and FOXP3^+^, which are mainly located in the GCs (Kim et al., 2017; Fonseca and Graca, 2019). Tfr cells can secrete IL-10 that acts as an immunosuppressant and maintains immunological tolerance. IL-10 also inhibits stimulation of B cells by Tfh cells (Chen et al., 2018). As an anti-inflammatory factor secreted by Tfh10 cells, IL-10 can effectively maintain intestinal balance. Cardoso et al. (2018) found that IL-10 can protect mice with DSS-induced colitis. Tfh17 cells produce IL-17, which induces autoimmune diseases and was increased in IBD, Guillain–Barré syndrome, Hashimoto’s thyroiditis, RA, systemic lupus erythematosus, and other diseases (Che, et al., 2016; Zhao et al., 2018; Sun et al., 2019). In the present study, the increase in Tfh17 cells was synchronous with the pathological colonic damage. It is important to note that SSP reduced the quantity of Tfh17 cells. Therefore, the balance between Tfr and Tfh10/Tfh17 cells is critical in the pathogenesis of autoimmune diseases (Wang et al., 2018). Higher numbers of Tfh17 or lower numbers of Tfh10 and Tfr cells can lead to inflammatory immunological damage in the colonic mucosa (Zhang et al., 2019). As shown in the present study, SSP can inhibit the decrease in Tfr and Tfh10 cells and the increase in Tfh17 cells to restore the balance of Tfr to Tfh10/Tfh17cells. These results suggest that SSP alleviates the pathological colonic injury induced by DSS, which is achieved by regulating the balance of Tfr and Tfh10/Tfh17 cells. However, the pathway regulating the balance is unclear.

Tfh cells, also known as CD4^+^CXCR5^+^ICOS^+^ T cells (Choi et al., 2011), have a regulatory effect on ICOS signaling during initiation of DCs in early Tfh development (Wikenheiser et al., 2016). ICOS can regulate activation of CD4^+^ T cells and support formation of GCs. Compared with ICOS^+/+^ mice, Tfh cells are reduced in the spleen of ICOS^−/−^ mice, which indicates that Tfh cell expansion is dependent on ICOS (Marriott et al., 2015; Wong et al., 2019). Furthermore, expression of ICOS is regulated by IL-6. In IL-6 receptor (IL-6R) knockout mice, expression of ICOS in Tfh cells is decreased, and sufficient IL-6R can provide an advantage for Tfh cell accumulation (Leavenworth et al., 2015). ICOS can induce differentiation of Tfh cells through the interaction of the p85α-OPN-i axis and Bcl-6 (Kolenbrander et al., 2018).

Recent studies have shown that the Bcl-6/Blimp-1 signaling pathway regulates Tfh cell differentiation and does not produce Tfh cells in the absence of Bcl-6 signaling. It is known that Bcl-6 is the regulatory transcription factor of Tfh cells (Wu et al., 2018). Just as Blimp-1 is an antagonist of Bcl-6, CD4^+^ T cells lacking Blimp-1 can preferentially develop into Tfh cells. High expression of Blimp-1 inhibits activation of Bcl-6, thereby inhibiting differentiation of Tfh cells (Linterman et al., 2010). Similarly, IL-21R can promote the expression of Bcl-6 (Choi et al., 2013; Lahmann et al., 2019). TGF-β, IL-6, and IL-6R can mediate STAT3-induced Bcl-6 expression and promote Tfh differentiation in human (Wu et al., 2015). In STAT3 deficiency, Tfh cells develop at a slower rate, inhibit IL-21, and increase expression of IFN-γ and IL-4, leading to decreased helper activity in B cells (Park et al., 2017; Deng et al., 2018). PD-1 is a co-inhibitory molecule that is highly expressed on Tfh cells, whereas PD-1^+^ Tfh cells play an important role in normal immune responses (Li et al., 2018; Shi et al., 2018). As a major marker of Tfh cells in GCs, PD-1 can inhibit T cells from entering follicles, allowing these T cells to concentrate in the GC and, together with PD-ligand 1 (PD-L1), optimize the competitiveness and affinity of B cells (Rajabian et al., 2019). The relative gene expression levels of PD-L1 and PD-L2 in UC patients are higher than those in the control group (Hu et al., 2013; Zhao et al., 2018). While SAP binding to SLAM family receptors increases cytokine secretion and regulates the duration of T and B cell interaction, enhancing Tfh cells’ help to GC and B cells, and leading to GC response defects in the case of SAP loss (Hu et al., 2013, 2016; Wang et al., 2015). In the present study, the percentages of ICOS^+^, BCL-6^+^, PD-1^+^, and PD-L1^+^ Tfh cells were downregulated in the peripheral blood of colitis mice treated by SSP, while the number of Blimp-1^+^ Tfh cells was upregulated. Synchronously, expression of BCL-6, p-STAT3, and SAP proteins in colonic mucosa was markedly decreased, and Blimp-1 activation was inhibited after 7-days’ treatment with SSP. Combined with the above analysis, we found that the BCL-6/Blimp-1 signaling pathway played an important role in the pathogenesis of experimental colitis induced by DSS; SSP inhibited BCL-6, p-STAT3, and SAP protein activation; and activated Blimp-1 expression to regulate Tfh cell differentiation. SSP restrained ICOS, Bcl-6, PD-1, and PD-L1 expression and activated Blimp-1 on the Tfh cell surface to limit the differential direction of Tfh cells, which improved the numbers of Tfh10 and Tfr cells and suppressed the number of Tfh17 cells to restore the balance of Tfh10 to Tfr/Tfh17 cells. All the above results combined with pathological analysis show that SSP eliminated the pathogenic immunocomplex in colonic mucosa by controlling the balance of Tfh10–Tfr/Tfh17 cells to restore immune stability and attenuate pathological colonic injury. However, it is regretful that we cannot analyze the levels of these protein molecules in the Tfh cells for absent fluorescence-activated cell sorting (FACS) Flow Cytometer. The results hinted SSP perhaps could regulate the expression of these protein molecules to create the environment of the differentiation of tissue resident Tfh cells from mesenteric lymph nodes and/or lamina propria to indirectly influence subpopulation of Tfh cells in peripheral blood.

In conclusion, the present study indicated that SSP regulated differentiation and function of Tfh cells to treat IBD, which was potentially related with inhibiting the Bcl-6/Blimp-1 pathway. To explore the explicit mechanism of SSP regulated differentiation of Tfh *via* BCL-6/Blimp-1 signaling pathway in the next studies, we will induce colitis animal model in the BCL-6 knock-in mice or Blimp-1 knock-out mice, separate tissue resident Tfh cells from mesenteric lymph nodes, and analyze the activation of Bcl-6/Blimp-1 signaling pathway after colitis mice were treated by Sishen Pill or Bcl-6 inhibitor or siRNA technology. As a traditional Chinese medicine, the effective constituents of SSP are extraordinarily complex. It is unknown which effective constituents of SSP optimize the function and differentiation of Tfh cells. In future work, we aim to establish the effective target for SSP regulation of Tfh cells to treat IBD by network pharmacology, metagenomics, and bioinformatics.

## Data Availability Statement

All datasets generated for this study are included in the article/supplementary material.

## Ethics Statement

The animal study was reviewed and approved by Institutional Animal Care and Use Committee (IACUC) of Jiangxi University of Traditional Chinese Medicine.

## Author Contributions

D-YL and H-MZ conceived and designed the experiments. X-KL, H-MZ, H-YW, WG, Y-BZ, and JL performed the experiments. D-YL and H-MZ contributed reagents, materials, and analytical tools. D-YL and X-KL analyzed the data. X-KL, H-MZ, and D-YL wrote the paper.

## Conflict of Interest

The authors declare that the research was conducted in the absence of any commercial or financial relationships that could be construed as a potential conflict of interest.
